# Supplementary material for: Unravelling metabolic cross‐feeding in a yeast–bacteria community using 13C‐based proteomics
Source: Mol Syst Biol. 2023 Feb 13;19(4):e11501. doi: 10.15252/msb.202211501 (PMC10090948; doi:10.15252/msb.202211501)
Supplement: Supplementary file 1 — Expanded View Figures PDF [file MSB-19-e11501-s005.pdf]

Expanded View Figures

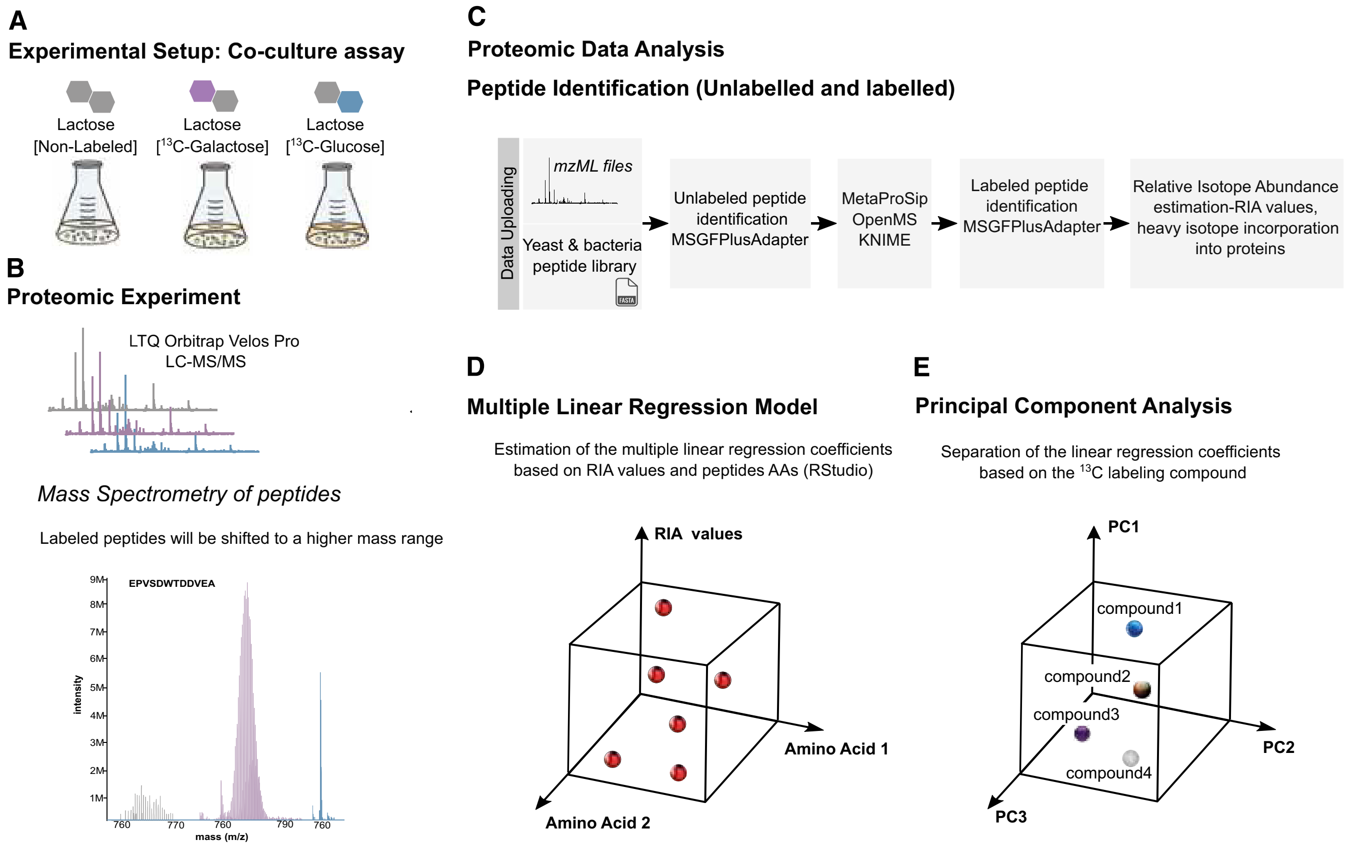

Figure EV1. Detailed workflow diagrams.

A Proteomics samples were collected from co-cultures grown in CDM35-lactose media with three different labelling regimes: non-labelled lactose, lactose with the galactose moiety labelled and lactose with the glucose moiety labelled.

B Tryptic peptides from all three labelling regimes were analysed on an LTQ Orbitrap Velos Pro instrument and the mass shift and relative isotope abundance values were determined from MS1 spectra.

C Overview of data analysis pipeline.

D Multiple linear regression (MLR) was used to determine the contribution of individual amino acids to peptide labelling in the different conditions.

E PCA was used for a global comparison of MLR coefficient profiles across different labelling regimes.

 © 2023 The Authors

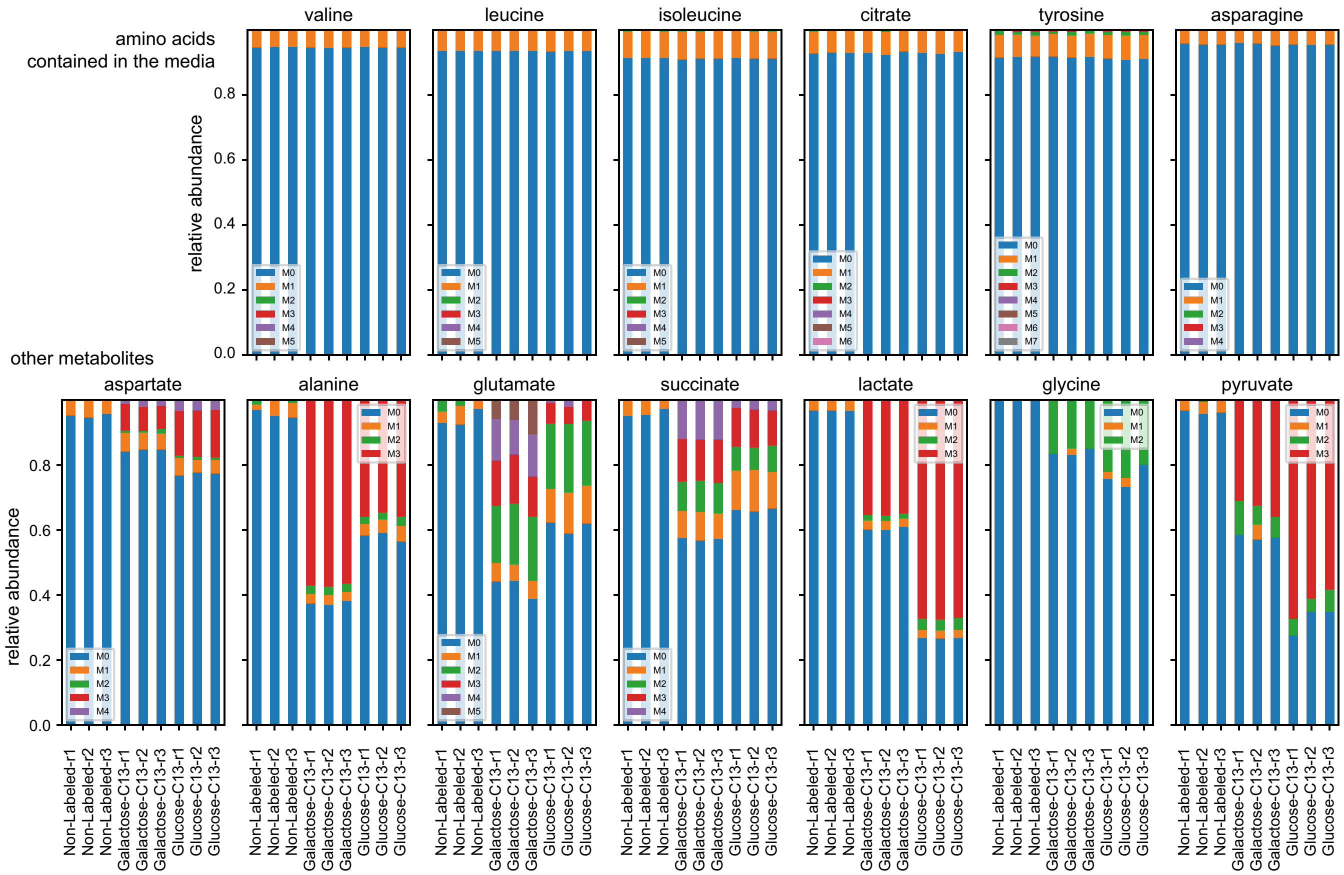

Figure EV2. Illustration of labelling states of extracellular metabolites in culture supernatant under three different labelling regimes.

Three biological replicates are shown individually.

© 2023 The Authors

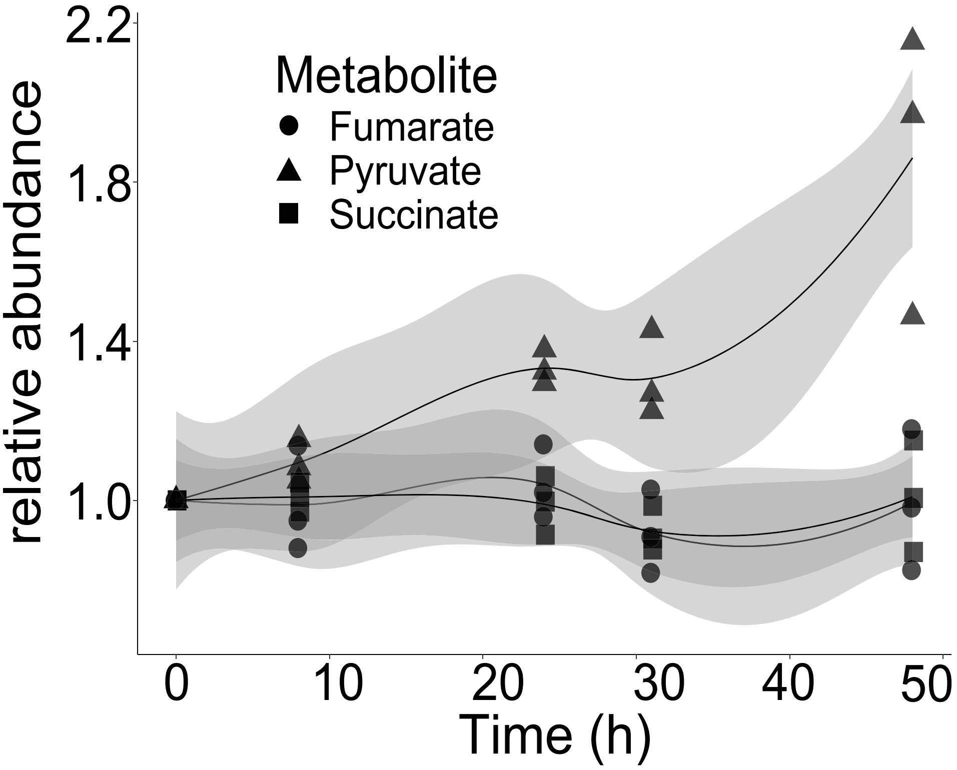

Figure EV3. Concentration dynamics of three metabolites during growth of *S. cerevisiae* on bacteria-conditioned media.

(n = 3 biological replicates)

Figure EV4. Composition of CDM42- and CDM35-lactose media.

CDM42-lactose contains many amino acids, supports growth of *L. lactis* monocultures and was used to generate bacteria-conditioned medium. CMD35-lactose contains fewer amino acids, does not support *L. lactis* growth and was used for co-culture experiments as it results in a mutualistic co-culture where neither species can grow alone.

 © 2023 The Authors

	Component	Concentration, g/L CDM42-lactose	CDM35-lactose
**C-source**	Lactose	40	40
**Amino Acids**	L-Glutamic acid	0.6	**0**
	L-Aspartic acid	0.05	**0**
	L-Glutamine	0.6	**0**
	L-Serine	0.5	**0**
	L-Threonine	0.5	**0**
	L-Alanine	0.4	**0**
	L-Glycine	0.3	**0**
	L-Arginine	0.72	0.72
	L-Asparagine	0.5	0.5
	L-Histidine	0.17	0.17
	L-Isoleucine	0.24	0.24
	L-Leucine	1	1
	L-Methionine	0.125	0.125
	L-Tyrosine	0.3	0.3
	L-Valine	0.7	0.7
**Vitamins**	Biotin	0.006	0.006
	Ca pantothenate	0.0012	0.0012
	Folic acid	0.00056	0.00056
	myo-Inositol	0.002	0.002
	Nicotinic acid	0.0009	0.0009
	p-Aminobenzoic acid	0.000056	0.000056
	Pyridoxine HCl	0.0048	0.0048
	Riboflavin	0.0009	0.0009
	Thiamine HCl	0.00056	0.00056
**Inorganic Salts**	$FeSO_4 \cdot 7H_2O$	0.005	0.005
	K_2HPO_4	6.48	6.48
	KH_2PO_4	3.12	3.12
	$MgCl_2$	0.3864	0.3864
	NaCl	3	3
	$ZnSO_4$	0.005	0.005
	K_2SO_4	0.023	0.023
	Boric acid	0.00075	0.00075
	$CaCl_2$	0.03	0.03
	$CoCl_2 \cdot 6H_2O$	0.00019	0.00019
	$CuSO_4$	0.00012	0.00012
	KI	0.00011	0.00011
	$MnSO_4 \cdot H_2O$	0.00034	0.00034
	$(NH_4)6Mo_7O_{24} \cdot 4H_2O$	0.00019	0.00019
**Other**	Ammonium citrate dibasic	1.69	1.69
	Citric acid$\cdot H_2O$	0.003	0.003
	L-Glutathione reduced	0.015	0.015
	pH	7	7

Figure EV4.

© 2023 The Authors
