# Supplementary material for: Unravelling metabolic cross‐feeding in a yeast–bacteria community using 13C‐based proteomics
Source: Mol Syst Biol. 2023 Feb 13;19(4):e11501. doi: 10.15252/msb.202211501 (PMC10090948; doi:10.15252/msb.202211501)
Supplement: Supplementary file 6 — PDF+ [file MSB-19-e11501-s001.pdf]

Article

TRANSPARENT PROCESS OPEN ACCESS

molecular systems biology

Unravelling metabolic cross-feeding in a yeast–bacteria community using ^{13}C-based proteomics

Natalia Gabrielli[1,†], Christoniki Maga-Nteve[1,†], Eleni Kafkia[1,2], Mandy Rettel[1], Jakob Loeffler[1], Stephan Kamrad[2], Athanasios Typas[1] & Kiran Raosaheb Patil[1,2,*]

Abstract

Cross-feeding is fundamental to the diversity and function of microbial communities. However, identification of cross-fed metabolites is often challenging due to the universality of metabolic and biosynthetic intermediates. Here, we use ^{13}C isotope tracing in peptides to elucidate cross-fed metabolites in co-cultures of *Saccharomyces cerevisiae* and *Lactococcus lactis*. The community was grown on lactose as the main carbon source with either glucose or galactose fraction of the molecule labelled with ^{13}C. Data analysis allowing for the possible mass-shifts yielded hundreds of peptides for which we could assign both species identity and labelling degree. The labelling pattern showed that the yeast utilized galactose and, to a lesser extent, lactic acid shared by *L. lactis* as carbon sources. While the yeast provided essential amino acids to the bacterium as expected, the data also uncovered a complex pattern of amino acid exchange. The identity of the cross-fed metabolites was further supported by metabolite labelling in the co-culture supernatant, and by diminished fitness of a galactose-negative yeast mutant in the community. Together, our results demonstrate the utility of ^{13}C-based proteomics for uncovering microbial interactions.

Keywords metabolic flux analysis; microbial interactions; protein stable-isotope labelling; synthetic microbial community
Subject Categories Biotechnology & Synthetic Biology; Metabolism; Microbiology, Virology & Host Pathogen Interaction
DOI 10.15252/msb.202211501 | Received 8 December 2022 | Revised 24 January 2023 | Accepted 25 January 2023 | Published online 13 February 2023
Mol Syst Biol. (2023) 19: e11501

Introduction

Prevalence of metabolite exchange in natural microbial communities is widely supported by bioinformatic as well as experimental evidence (Henriques *et al*, 2020; Blasche *et al*, 2021; Giri *et al*, 2021; Machado *et al*, 2021; Estrela *et al*, 2022; Pontrelli *et al*, 2022; Yu *et al*, 2022). The latter, however, is much more limited, both in terms of the coverage of the communities and the depth of the evidence. Most experimental evidence stems from associations between nutritional perturbations and community compositional dynamics, with few studies pinpointing the exchanged metabolites and the corresponding donors and receivers. A major challenge is the universality of central metabolites, for example, amino acids, nucleotides and organic acids, across the tree of life. Unlike DNA and RNA, these metabolites cannot be assigned to a species once outside the cell. While genomic auxotrophies can suggest exchanged metabolites in natural communities (Zelezniak *et al*, 2015; Giri *et al*, 2021) and provide a basis for engineering synthetic communities (Chang *et al*, 2021; Guillen *et al*, 2021; Konstantinidis *et al*, 2021), even then the identification of the exchanged metabolites can be difficult due to the complexity of the metabolic networks. Cellular regulation can switch off certain biosynthetic pathways in an environment-dependent manner, and many different metabolites can compensate for a given auxotrophy. While some of the obstacles can be alleviated by (meta-) transcriptomics/proteomics measurements, their utility in predicting metabolite secretion is limited even in single species studies. Direct metabolic measurements are surprisingly uncommon, even for synthetic communities, and thus, the list of experimentally validated inter-species metabolic interactions is small. Thus, mechanistic understanding of community dynamics in response to, for example, environmental change, as well as rational modulation of microbial communities, is currently limited.

Metabolic flux analysis using stable isotope tracing has been successfully used for resolving the fate of nutrients in single species (Wiechert, 2001; Schwechheimer *et al*, 2018; Xiao *et al*, 2022). However, the analysis methods, which are based on proteogenic amino acids or direct metabolite measurements, cannot be directly applied to communities as most metabolites cannot be ascribed to the producer/consumer member species. An alternative approach is to separate DNA extracted from the community by mass, which is indicative of the degree of mass-isotopomer incorporation; subsequent sequencing can then identify the community members that incorporate a labelled substrate (Radajewski *et al*, 2000; Bernard *et al*, 2007). Similarly, the protein stable isotope probing (Protein-SIP) technique (Jehmlich *et al*, 2010, 2016; von Bergen *et al*, 2013;

1 European Molecular Biology Laboratory, Heidelberg, Germany
2 Medical Research Council Toxicology Unit, University of Cambridge, Cambridge, UK
*Corresponding author. Tel: +44(0)1223335640; E-mail: kp533@cam.ac.uk
†These authors contributed equally to the work

© 2023 The Authors. Published under the terms of the CC BY 4.0 license.

Sachsenberg *et al*, 2015) has been used to unambiguously identify the peptides and the metabolically active phylogenetic groups in the community (Jehmlich *et al*, 2008). This requires tailored mass spectrometry data analysis methods that simultaneously deconvolute potential mass shifts and species identities (Sachsenberg *et al*, 2015, Jehmlich *et al*, 2016). Both the DNA and protein-based methods, as well as similar approach to lipid labelling have been key to tracing mass-flow and identifying key players in hydrocarbon-degrading communities (Bastida *et al*, 2010; Herbst *et al*, 2013; Vogt *et al*, 2016). Yet, while these methods can identify direct utilizers of the labelled substrates and potential interactors, intra-cellular flux distributions and cross-fed metabolites cannot be easily resolved. Towards resolving intra-cellular fluxes, the ^{13}C labelling combined with meta-proteomics can in principle be used to infer labelling at the level of amino acids within community members if enough peptides can be uniquely assigned (Ghosh *et al*, 2014; Gebreselassie & Antoniewicz, 2015; Sachsenberg *et al*, 2015). However, this concept has, to our knowledge, remained experimentally untested beyond engineered conspecific strains (Gebreselassie & Antoniewicz, 2015).

Here we used a yeast–lactic acid bacterial community to evaluate the peptide labelling strategy for elucidating inter-species metabolic exchange. While of much lower complexity than naturally occurring microbial communities, this two-member community is an excellent model to develop approaches to tackling this highly complex experimental problem. Yeast *Saccharomyces cerevisiae* and lactic acid bacteria such as *Lactococcus lactis* co-exist in many natural and fermented food environments such as sourdough, kefir and wine (Ferreira & Mendes-Faia, 2020; Blasche *et al*, 2021; Pino *et al*, 2022). These species are thus important for ecology as well as widely used as model organisms. We have previously shown the critical role of metabolic cross-feeding in this symbiosis and identified nitrogenous compound overflow (esp. amino-acid) as a key mechanism of lactic acid bacteria (LAB) support by yeast (Ponomarova *et al*, 2017). A notable feature of this community is that it can be readily assembled in a defined medium and the one-way (commensal) relation can be turned into mutualism by changing a single nutrient, namely glucose to lactose. In the mutualistic scenario, yeast, being unable to breakdown lactose, depends on LAB for carbon, while the LAB relies on the yeast for nitrogen. While the latter is known to be mediated by amino acids, a key open question is which metabolites are shared by the lactic acid bacteria with the yeast (Fig 1A). We here answer this question by using ^{13}C label-tracing in peptides and extracellular medium, providing first proof of concept for using proteomics for inferring inter-species metabolic interactions.

Results

Assembly of a mutualistic community of *S. cerevisiae* and *L. lactis*

We assessed the capacity of several lactic acid bacteria to form a mutualistic community with *S. cerevisiae* on a defined medium containing lactose and 8 different amino acids. Two *L. lactis* strains isolated from the fermented milk drink Kefir (Blasche *et al*, 2021), but not the *L. lactis*-type strain IL1403, stained positive for beta-galactosidase activity and formed characteristic 'crescent moon' shapes when pinned next to *S. cerevisiae* (Fig 1B). Strain SB261 was chosen for all further experiments and showed robust growth on lactose, glucose, a glucose-galactose mix, but not galactose alone (Fig 1C). The media used in all following experiments was chosen so that the two species grow together but neither *L. lactis* nor *S. cerevisiae* could grow alone due to their respective requirements for amino acids and carbon from the other species (Fig 1D).

^{13}C incorporation analysis in co-cultures by proteomics

Some LAB strains harbour galactose-lactose antiporters and thus export galactose in the presence of lactose (Hutkins & Ponne, 1991; Benthin *et al*, 1994). To probe whether galactose is the carbon source shared by *L. lactis* with yeast, we performed co-culture proteomics analysis using ^{13}C-labelled lactose (Fig EV1). Yeast and *L. lactis* were grown together with either non-labelled lactose, lactose labelled in the carbons corresponding to the glucose backbone lactose-[^{13}C-glucose], or lactose labelled in the carbons corresponding to the galactose backbone lactose-[^{13}C-galactose].

The mass spectrometry data from the co-cultures were analysed using MetaProSIP (Sachsenberg *et al*, 2015) tool within the OpenMS framework (Rost *et al*, 2016; Fig 2A). The assignment of labelled and unlabelled peptides to bacteria or yeast was based on the reference peptides from non-labelled lactose sample corresponding to 2,693 and 6,721 proteins respectively (Dataset EV1). A target decoy database including peptides from other organisms was used to detect any incorrect assignments of isotope-labelled peptides. Among the 49,365 peptides identified in the samples, only 279 were deemed false positives, indicating the incorporation false positive rate (iFPR; Griss *et al*, 2014; Sachsenberg *et al*, 2015) to be circa 0.005 (Dataset EV1). We estimated the degree of incorporated ^{13}C atoms within each peptide in terms of Relative Isotope Abundance (RIA, Materials and Methods, Dataset EV2). Both yeast and bacterial proteomes showed good reproducibility in RIA values across peptides (Fig 2B) indicating overall uniformity in isotope incorporation.

Peptide-labelling patterns reveal galactose as a metabolite shared by *L. lactis*

When the lactose was labelled in the galactose subunit, the labelling degree in the yeast peptides was considerably higher (~ 30%, we note that the growth medium contains unlabelled amino acids) than when the glucose subunit was labelled (~ 5%; Fig 2B). This difference indicates that the bacteria, upon lactose breakdown, preferentially use glucose, while galactose is secreted and taken up by yeast. In bacterial proteome, the pattern was expectedly reversed with degree of labelling being higher when lactose-[^{13}C-glucose] was fed (~ 19%) than in the case of lactose-[^{13}C-galactose] (~ 15%; Fig 2A). This suggests that lactose is metabolized intracellularly by *L. lactis* and galactose is released, as with other dairy lactococci. (Leenhouts *et al*, 1995). The relatively small difference in the labelling degree between two substrates, as well as the overall smaller labelling than yeast, is consistent with the bacterial reliance on amino acids in the growth medium and those secreted by the yeast. The yeast cells, on the other hand, channelled more carbon from the galactose to protein synthesis. Our data do not exclude that a small fraction of the galactose is metabolized by *L. lactis*, although its poor growth on this substrate (Fig 1C) and preference for glucose utilization makes this unlikely to be at an appreciable level.

 © 2023 The Authors

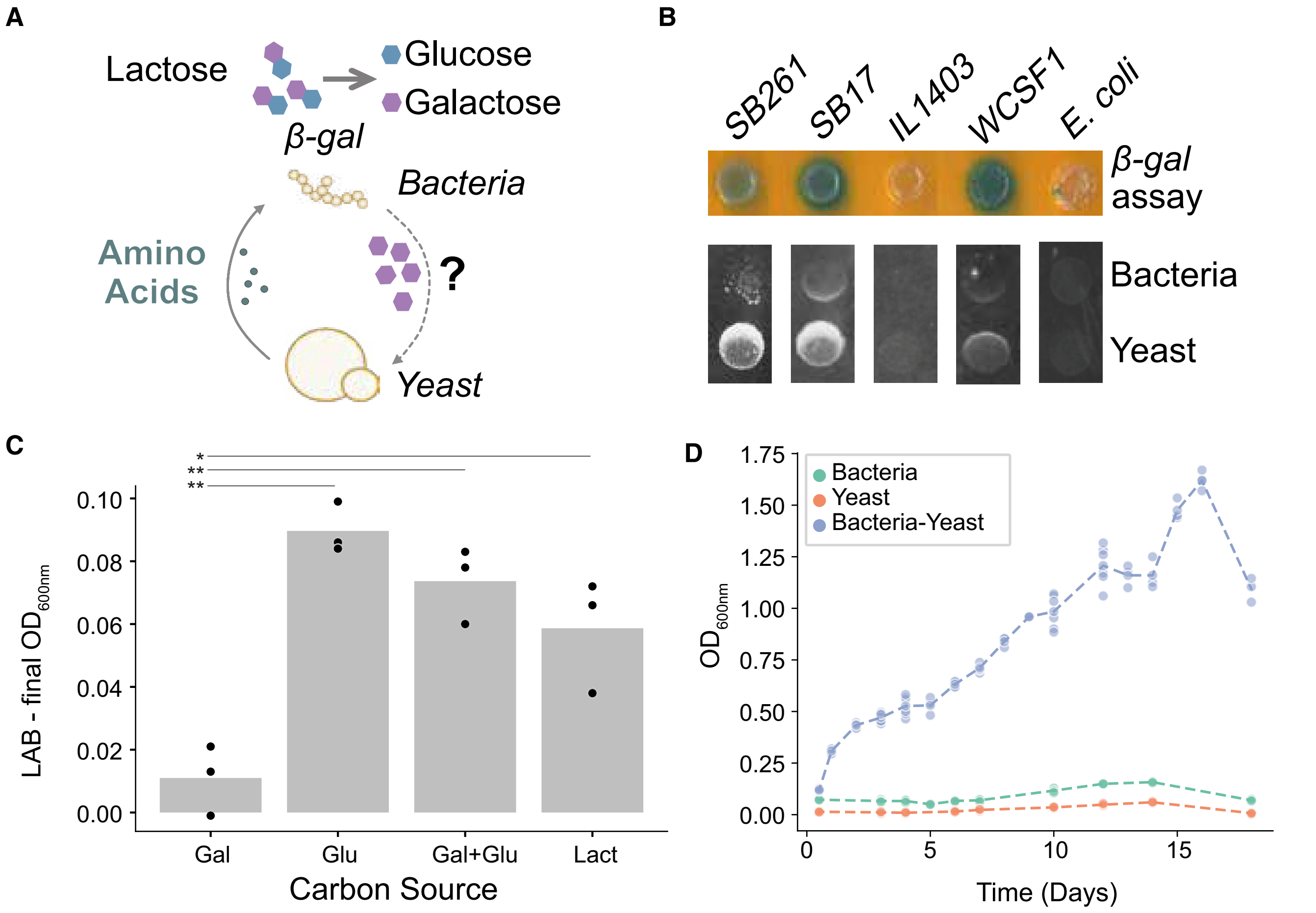

Figure 1. Lactic acid bacteria and *S. cerevisiae* enable mutual growth.

A Schematic hypothesis of metabolite exchanges in the microbial community.

B Screen of LAB strains and *E. coli* for beta-galactosidase activity (top) and growth in close proximity to *S. cerevisiae* colonies on CDM35-lactose plates (bottom). Strains SB261 and SB17 are *L. lactis* strains isolated from Kefir in a previous study (Blasche *et al*, 2021); IL1403 and WCSF1 are *L. lactis* and *Lactiplantibacillus plantarum* type strains respectively. The "crescent moon" shape observed in spots with SB261, SB17 and WCSF1 indicates that both yeast and LAB benefit from proximity to each other, that is, indicate the potential to form a cooperative co-culture. Strain SB261 was selected to be used in all following experiments.

C Bacterial growth (OD_{600}) of *L. lactis* strain SB261 in different carbon sources after 26 h. Bar heights indicate the mean. Values for each carbon source were compared to growth in galactose and the statistical significance is indicated ($n = 3$ biological replicates, two-sided Student's *t*-test, *P*-values: glucose: 0.00059; glucose + galactose: 0,0.0027; lactose 0.018).

D Dynamics of *L. lactis* SB261 growth in co-culture with *S. cerevisiae* WT S90 strain (blue line) and both species in monoculture (green and orange lines) in CDM35-lactose media. The lines show the mean of three biological replicates.

To trace the flow of labelled carbon from lactose to the yeast and bacterial peptides, we estimated the contribution of individual amino acids to peptide RIA values using multiple linear regression (Materials and Methods). This allowed us to estimate the average, relative, contribution of individual amino acids to the mass shift/labelling state of peptides, specific to each species and labelling regime (galactose or glucose moiety labelled). (Dataset EV2). Principal component analysis (PCA) showed clear distinction of the yeast-lactose-[^{13}C-galactose] in accord with the highest labelling incorporation seen from overall RIA distribution (Fig 2C). At the level of individual amino acids, the contribution of amino acids provided in the growth medium to the labelling was minimal as expected, while those secreted by yeast (glycine, alanine, glutamate, glutamine, serine, threonine and aspartate; Ponomarova *et al*, 2017) had the highest contribution (Fig 2C and D). Three amino acids with a close link to the central carbon metabolism, viz., alanine, glycine and cysteine, had stronger labelling contribution under lactose-[^{13}C-galactose] growth, attesting the entrance of ^{13}C in yeast via glycolysis. The dependency of *L. lactis* on the yeast-supplied amino acids was also well reflected in the bacterial peptide labelling (Fig 2D). The overall distribution of individual amino acid contributions in bacterial peptides was thus similar to that of yeast (Fig 2C and D). The activity of the galactose utilizing Leloir pathway in yeast was evident in the expression of Gal1, Gal2, Gal3, Gal7, Pgm1/2 and Gal10 in the unlabelled proteomics data set (Dataset EV2). Together, the labelling patterns reveal galactose produced by *L. lactis* as

© 2023 The Authors

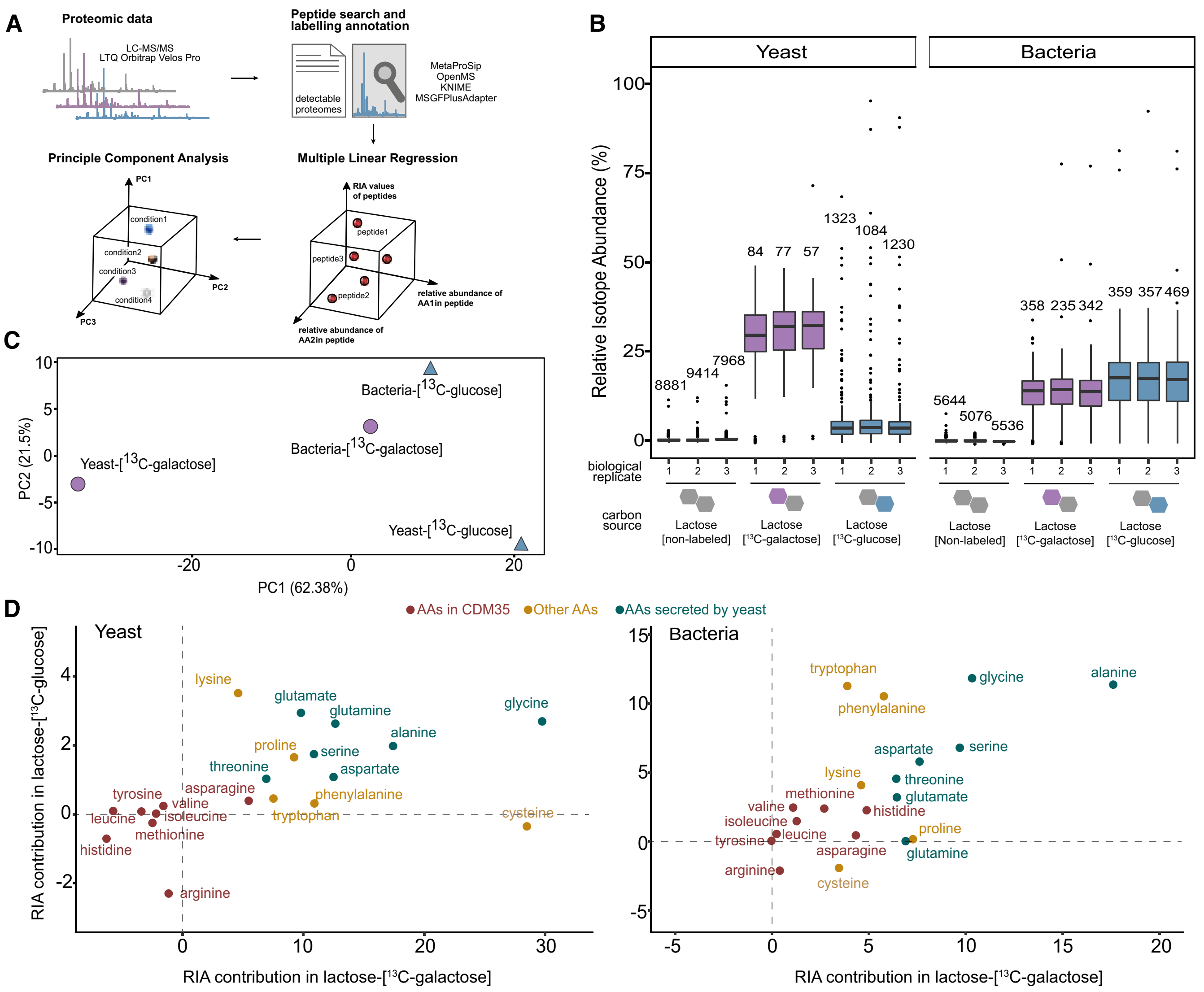

Figure 2. Analysis of labelling patterns in species-specific peptides by proteomics identifies cross-fed amino acids.

A Proteome data were acquired for yeast–LAB communities grown on lactose with either the glucose or galactose part labelled. The MetaProSIP workflow was used to identify peptides, their species identity, and relative isotope abundance (RIA, fraction of carbons labelled). The contribution of individual amino acids to peptide labelling was estimated using multiple linear regression. The resulting coefficient vectors for the four species–substrate pairs were then compared using Principle Component Analysis (PCA).

B Box plots of RIA distributions in the co-culture assay. All peptides identified for each species were grouped based on their biological replicates and discriminated by the labelling conditions. Numbers above the boxes indicate the number of peptides in this group. As is standard, this boxplot shows the median of the data as central line, the quartiles as box and the extend of the rest of the distribution as whiskers. Points which are 1.5 times the inter-quartile range beyond the high and low quartiles are considered outliers and shown individually.

C PCA discriminates the yeast-lactose-[^{13}C-galactose] from the other three groups.

D Predicted estimated coefficients relationship between the lactose-[^{13}C-galactose] and lactose-[^{13}C-glucose] in yeast (left) and bacteria (right) based on 3 different AA groups. Higher values indicate more ^{13}C labelling.

the main carbon source of yeast and the reciprocity of yeast through amino acids that enter bacterial biomass.

Exo-metabolome reveals a multi-factorial cross-feeding

The labelling enrichment in yeast peptides under lactose-[^{13}C-glucose] compared to unlabelled control indicates that yeast receives bacterial metabolites other than galactose. To identify these metabolites, and to affirm the peptide-based inference of cross-fed metabolites, we used GC–MS to analyse ^{13}C labelling in the metabolites in the co-culture supernatants (Materials and Methods, Dataset EV3). Circa 70% of pyruvate and lactate were found to be labelled in all carbon atoms during growth on lactose-[^{13}C-glucose], but only 40% in the case of lactose-[^{13}C-galactose] supplementation

© 2023 The Authors

(Fig 3). This is consistent with the bacteria being the main utilizers of glucose and lactic acid being a main fermentation product of *L. lactis* metabolism. Pyruvate, which is only a single enzymatic step away from lactic acid, had a singular labelling pattern as lactic acid. Furthermore, co-culture proteomics data showed presence of lactate dehydrogenase (Dld1p, detected in 3/3 unlabelled samples) consistent with bacterial secretion of both lactate and pyruvate into the medium.

Amino acids that were added to the medium showed, as expected, little ^{13}C labelling (Fig EV2). Among the other amino acids, glycine and aspartate had approximately 15–20% fractions labelled but did not show appreciable difference whether the lactose was labelled in galactose or glucose. Both amino acids thus appear to have been synthetized and secreted by yeast as well as *L. lactis*. Alanine had 20% higher labelling when the galactose part of lactose was labelled, attesting the proteomic results wherein the contribution of alanine to peptide labelling was high in lactose-[^{13}C-galactose] samples for both organisms.

Circa 60% of glutamate in the supernatant was labelled in at least one carbon atom in the case of lactose-[^{13}C-galactose] and 40% in the case of lactose-[^{13}C-glucose] indicating that carbon flow from both species and that from other, unlabelled, media components contributed to glutamate synthesis. Yet, the labelling pattern in terms of the number of labelled carbons was different between lactose-[^{13}C-galactose] and lactose-[^{13}C-glucose]. When lactose-[^{13}C-galactose] was fed, glutamate molecules had 4 or 5 carbons labelled; while when fed with lactose-[^{13}C-glucose], only 1, 2 or 3 carbon atoms were labelled. Succinate followed the same patterns as glutamate since it is a downstream metabolite. Glutamate thus clearly originated from different initial carbon sources or from different biosynthetic pathways between the two cases, that is, lactose-[^{13}C-galactose] versus lactose-[^{13}C-glucose]. *L. lactis* lacks a complete TCA cycle (Morishita & Yajima, 1995) and thus the difference can be, in part, ascribed to the glutamate originating from yeast (4–5 carbons labelled) or *L. lactis* (1–3 carbons labelled). Overall, the metabolite labelling in the co-culture supernatant corroborates the cross-feeding interactions revealed by proteomics. Together, these show that bacteria share, beyond galactose, pyruvate, lactic acid, glycine, aspartate and glutamate into the medium and contribute to carbon into yeast proteome, while yeast cells primarily share amino acids.

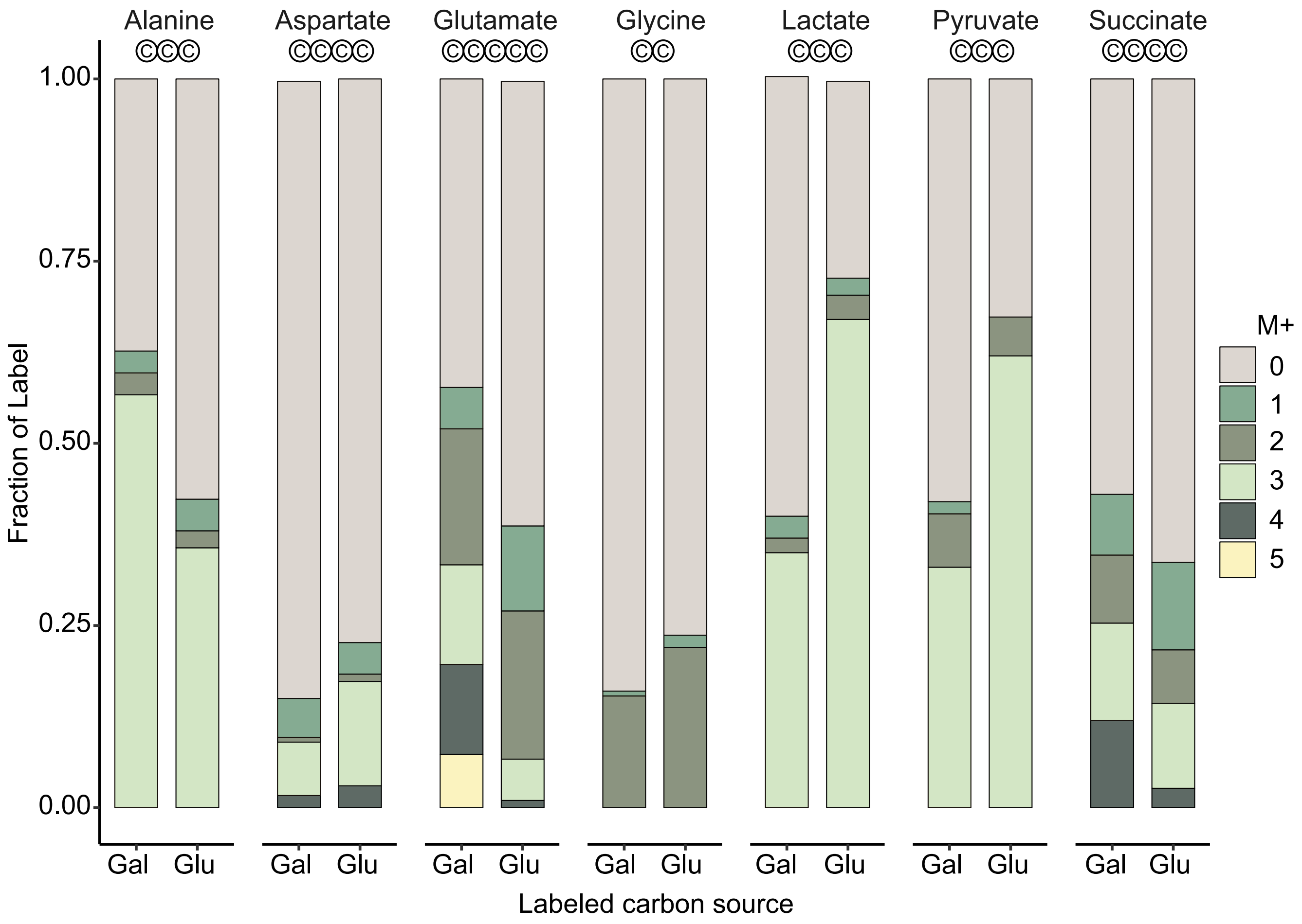

Figure 3. Comparing labelling of metabolites in exometabolome under different label supplementations reveals donor species of secreted metabolites.
Metabolites from the supernatants of the co-cultures feed with labelled lactose [^{13}C-galactose] or lactose [^{13}C-glucose] measured by GC–MS. The circles labelled ‘c’ under the metabolite name indicate the number of carbon atoms in the metabolite.

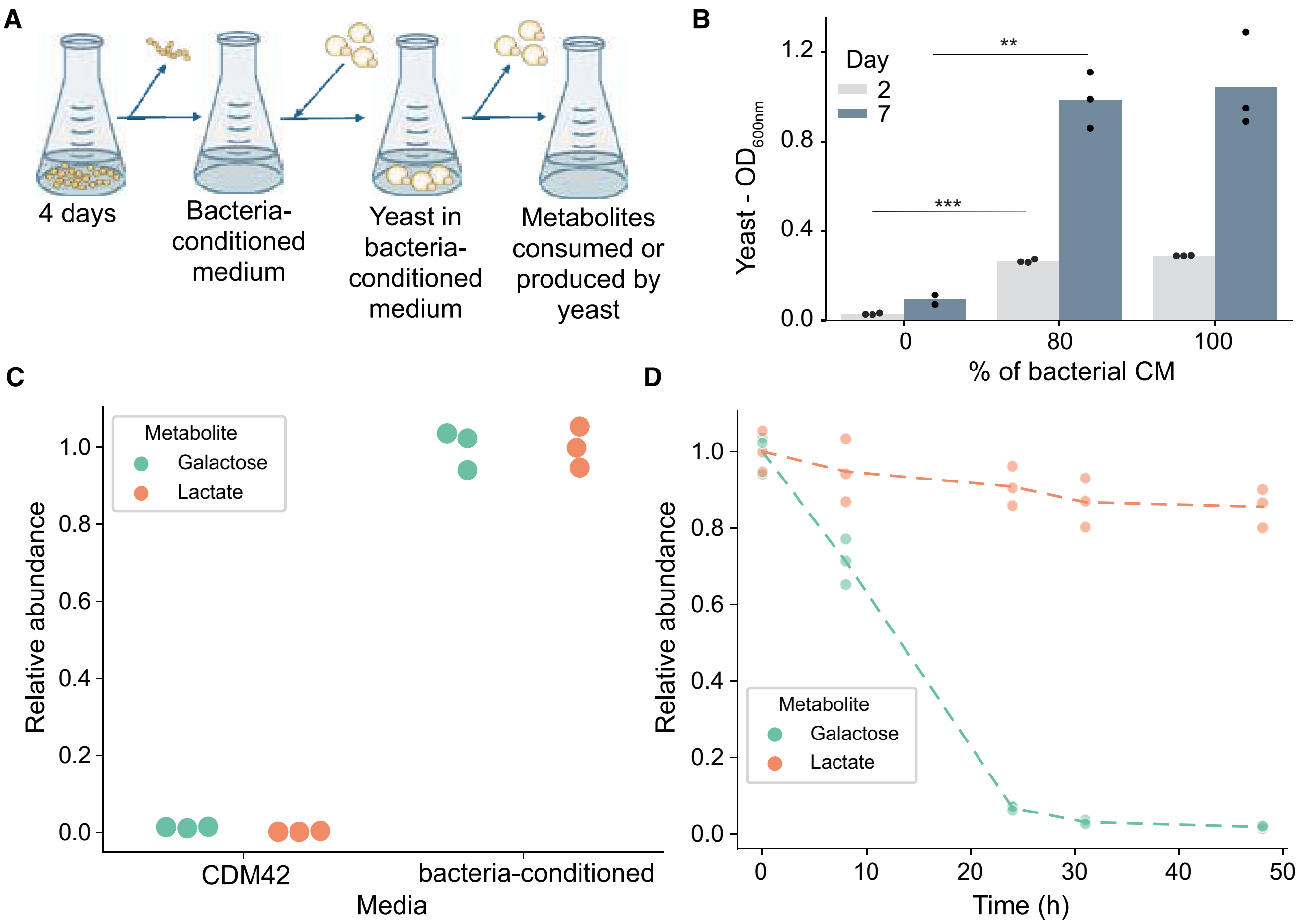

Figure 4. Identification of bacterial secretome components that enable yeast growth.

A Conditioned medium assay design.

B Bar graph representing yeast growth in different percentages of bacterial spent medium. Bar heights represent the mean of $n = 3$ biological replicates. The statistical significance of the difference in growth between 0 and 80% bacterial conditioned is indicated for days 2 and 7 (two-sided Student's *t*-test, *P*-values: day 2: 1.27×10^{-6}; day 7: 0.0025).

C Targeted metabolomics using GC–MS applied to the bacterial conditioned medium, metabolites produced by LAB (relative to the mean concentration in bacteria-conditioned media). Data represent three biological replicates.

D Exo-metabolome dynamics revealed by GC–MS targeted metabolomics, metabolites that potentially cross-fed from bacteria to yeast, produced by LAB and consumed by yeast (see Dataset EV3 for the complete list of metabolites measured). Data represent three biological replicates, lines represent the mean.

Metabolic modelling, spent medium assay and gene knockout mutants complete the cross-feeding scenario

We next used genome-scale metabolic models to link the observed metabolic cross-feeding to genomic features, esp. auxotrophies, of the yeast and *L. lactis* strains (Materials and Methods). At the level of individual species, flux balance simulations confirmed the amino acid auxotrophies of *L. lactis* and the inability of yeast to utilize lactose. Community simulations confirmed the inter-dependencies of two species, particularly that of yeast on galactose and that of bacteria on amino acids secreted by yeast. The modelling also suggested other possible interactions mediated by aromatic amino acids or lactate (Materials and Methods, Dataset EV4).

To independently confirm the carbon flow between *L. lactis* and yeast, we cultivated yeast in bacterial spent medium (Fig 4A and B) and followed the metabolites in the supernatant using GC–MS (Dataset EV3). Galactose and lactate were the main components in the condition medium of which galactose had been consumed after 20 h while only a small reduction was seen in lactate after the galactose was exhausted (Fig 4C and D). Other metabolites in the bacterial secretome, such as fumarate, succinate and pyruvate, were not consumed by the yeast, while pyruvate was secreted (Fig EV3). The spent medium assay thus further confirmed bacteria-supplied galactose as the primary carbon source of yeast, yet also suggested that other metabolites like lactate and pyruvate could potentially support yeast growth. To genetically test this hypothesis, we replaced the wild-type yeast by *gal1Δgal3Δ* mutant, which is not able to utilize galactose (Fig 5B). The co-culture of the mutant yeast and *L. lactis* had a reduced fitness, yet the growth was not abolished, supporting our hypothesis (Fig 5C). Together, the peptide and supernatant

 © 2023 The Authors

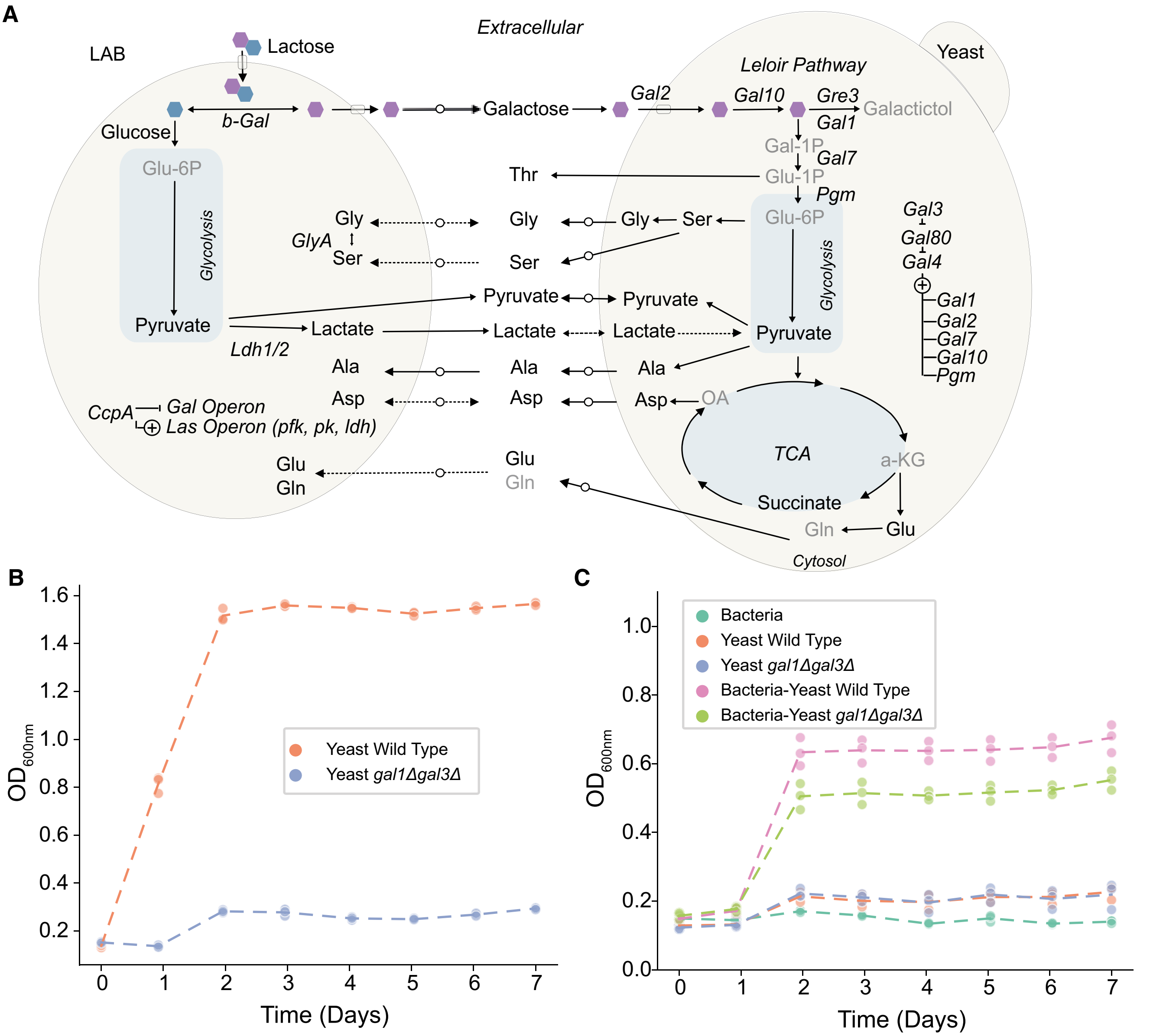

Figure 5. Galactose is the main mediator of the trophic interaction between LAB and yeast.

A Qualitative scheme of the metabolic interchange in the co-culture growing in CDM35-Lactose. Dashed lines indicate processes not conclusively supported by experimental evidence. Arrows with an overlaid circle represent processes supported by modelling.

B The yeast *gal3Δgal1Δ* deleted mutant is not able to grow in CDM35-galactose. Data represent three biological replicates, lines represent the mean.

C Co-culture growth is compromised but not completely impeded if the LAB co-cultured with yeast is unable to metabolize galactose. Co-cultures and mono-cultures were grown in CDM35-lactose medium. Data represent three biological replicates, lines represent the mean.

metabolite labelling patterns, modelling, spent medium assays, and *gal1Δgal3Δ* mutant community affirm galactose as a preferred but not the only carbon flow from bacteria to yeast (Fig 5A).

Discussion

Metabolite cross-feeding emerges from overflow metabolism and complementary metabolic requirements of interacting species (Paczia *et al*, 2012; Campbell *et al*, 2015, 2018; Ponomarova *et al*, 2017; Yu *et al*, 2022). Metagenomic analyses can thus be used to map potential metabolic interactions through identification of metabolic pathways that are genetically absent (Zelezniak *et al*, 2015; Machado *et al*, 2021). However, genetic auxotrophies can underestimate the number of metabolic interactions as community members can secrete many metabolites forming "common goods". Several members then may not utilize all genetically encoded metabolic functions when a metabolite (or its precursor) is available in their environment (Ljungdahl, 2009; Bianchi *et al*, 2019). Transcriptomic and proteomics analyses can make

© 2023 The Authors

some contribution towards understanding the set of expressed metabolic genes, but ultimately fall short as enzyme levels are not easily relatable to metabolic fluxes, and even less so for the import/export of metabolites from the environment (Hackett *et al*, 2016; Sauro, 2017; Zelezniak *et al*, 2018). Direct experimental evidence of metabolite sharing is therefore required to establish the molecular interaction network, but so far is only sparsely available.

One approach has extended classical metabolic flux analysis (MFA) by linking two metabolic models with exchange fluxes and fitting intra- and inter-cellular flux distributions using mass isotopomer distributions (MIDs) obtained from peptide labelling data. But so far, these studies were either within a species (Gebreselassie & Antoniewicz, 2015) or theoretical (Ghosh *et al*, 2014). Other approaches to disentangling communal metabolism have used the incorporation of stable isotopes into proteins, albeit with the a limited aim of identifying species (groups) consuming/degrading a specific isotope-labelled substrate (Jehmlich *et al*, 2016) rather than metabolite exchange between cells.

We here built on the protein-SIP concept to capture cross-feeding interactions, providing a first experimental proof-of-concept for inferring interspecies metabolic exchange using isotope tracing in co-culture proteomics. The peptides provided the species identity while the isotope enrichment indicated the flux channelling; the method thus circumvented the limitations of classic metabolite-based flux analysis wherein the metabolites cannot be ascribed to the species of origin. Labelling analysis of metabolites in the co-culture supernatant agreed well with the peptide-based inference and helped to identify additional cross-fed metabolites. Metabolic modelling accounting for the total biosynthetic capabilities (or lack thereof) were also consistent with the findings. Importantly, few of the modelling predictions (e.g. tryptophan and phenylalanine) were not supported by the data. This is, however, expected, as while the models outline possible exchanges, enzyme expression and other operational or regulatory constraints are not accounted for, and are difficult to do so in a generalized fashion. Metabolite sharing is not a consequence of nutrient release by dead cells as label incorporation into protein depends on live, metabolically active cells. Furthermore, the community is stable over long timescales and can be evolved to enhance metabolite sharing (Konstantinidis *et al*, 2021), and the fraction of dead cells observed in yeast–LAB co-cultures is minimal (Ponomarova *et al*, 2017).

Yeast and lactic acid bacteria are quite distinct in their protein sequence space; in communities involving phylogenetically closely related species and/or those with larger membership, disentangling species identity and label incorporation will become difficult. Application of the peptide-based flux analysis to more complex communities would, therefore, require both theoretical and experimental development. Furthermore, the approach requires differential preference of carbon substrates. Yet, as our results exemplify, proteomics, metabolomics and metabolic modelling could be jointly used to disentangle complex metabolic interactions.

Materials and Methods

Strains, media and growth conditions

All cultures were grown statically at 30°C. Chemically defined medium, CDM42-lactose for bacteria-conditioned medium and CDM35-lactose for bacteria and yeast co-culture growth assays, were prepared based on the medium designed in our previous work (Fig EV4; Ponomarova *et al*, 2017). The lactose concentration in the medium was 4%. Labelled lactose was purchased from Omicron including [UL-$^{13}C_6^{glc}$]-lactose and [UL-$^{13}C_6^{gal}$]-lactose monohydrate (LAC-006, LAC-007). All solutions were sterilized by filtration. Liquid medium experiments were started at 0.1 OD_{600} for bacteria and 0.05 OD_{600} for yeast cultures, using pre-cultures washed twice with phosphate-buffered saline (PBS). We used CDM35-galactose liquid media (2% and 0.05%) and SD-galactose 2% agar plates to test the phenotype of the *gal1Δgal3Δ* double mutant. For assays on solid media, 5 µl of the washed LAB cultures (0.1 OD_{600}) and yeast culture (0.2 OD_{600}) were spotted on CDM35-lactose plates (2% agar). LAB cultures were placed in proximity to the yeast inoculum and further away on the same plate as a control. CDM35-lactose agar plates were incubated for 9 days. Cell growth was monitored by measuring the optical density at 600 nm (OD_{600}) using a spectrophotometer (Biochrom Ultrospec 2100 pro, or BioTek Synergy for growth assays in 96-well plates). Growth experiments were conducted in biological triplicates.

Yeast strain construction

The double knockout mutant *gal1Δgal3Δ* was constructed for this study in the prototrophic haploid laboratory S90 genetic background. Genomic DNA of a *gal1Δ::kanMX4* mutant in BY4741 background (Mulleder *et al*, 2012) was extracted and a fragment containing the kanMX4 cassette with 25 base pairs of flanking genomic regions was PCR amplified using previously described A and D primer pairs (Winzeler *et al*, 1999; here called PNG45_A and PNG47_D). Two deoxyoligonucleotides were designed based on the *gal3* nucleotide sequence and the multiple cloning site present in the pCfB2194 vector (Stovicek *et al*, 2015). To generate *gal3Δ::hphMX4* cassette the deoxyoligonucleotide PNG53 comprised 25 bp of the 5′ region of the *GAL3* 5UTR sequence (+603 to +628), and 22 bp of the pCfB2194 multiple cloning site. The deoxyoligonucleotide PNG57 contained 25 bp corresponding to the 3UTR *GAL3* gene (+2,836 to +2,861) and 20 bp from the pCfB2194 multiple cloning site. Qiagen-purified pCfB2194 DNA was used as template for PCR amplification. These PCR products were used to transform wild-type *S. cerevisiae* S90 as described previously (Gietz & Schiestl, 2007) with some modifications. A mid-log culture (OD_{600} = 0.7, 50 ml) was centrifuged, washed, and re-suspended in 1 ml of sterile water. Then 100 µl of cell suspension were mixed with transformation mix (240 µl PEG 3500, 50% w/v; 36 µl lithium acetate, 1.0 M; 50 µl boiled single-stranded carrier DNA, 2 mg/ml; 34 µl of PCR amplification product described above). After incubation for 40 min (42°C water bath), cells were re-suspended in YPAD and incubated for 3–4 h to allow for the expression of the integrated antibiotic marker. Clones were selected on YPAD plates with G418 (300 µg/ml) and subsequently on hygromycin B (Formedium Ref No: HYG1000) plates (200 µg/ml). Success of homologous recombination was verified by colony PCR using PNG46_C-PNG47_D, KanB-PNG45_A, PNG48_A-KanB and PNG49_C-PNG50_D primer pairs.

Conditioned medium assay

L. lactis SB261 was cultured in CDM42-lactose medium for 4 days (approx. 0.5 OD_{600}) at 30°C without shaking to create bacteria-

 © 2023 The Authors

conditioned medium. Yeast was grown for 7 days in 80% (supplemented with 20% of fresh medium) or 100% of bacteria-conditioned medium at 30°C without shaking. At indicated time points, 1 ml of cell suspension was sampled and centrifuged at 4,000 *g* at 4°C for 1 min. Supernatants were passed through 0.2 μm syringe filter PES (Millipore). Metabolites of the bacteria-conditioned medium and the subsequent medium after yeast growth were measured by GC–MS.

Targeted metabolomics analysis of extracellular spent growth media with GC–MS

Supernatants of bacterial monocultures in CDM42-lactose, supernatants of yeast in CDM42-lactose bacteria-conditioned medium and yeast–*L. lactis* co-cultures in CDM35 with labelled lactose were filtered with 0.2-μm PVDF syringe filters and 3-kDa MWCO filters (Millipore). Ribitol (Adonitol; Alfa Aesar, UK) was added as an internal standard at a final concentration of 50 μg/ml. Polar metabolites were extracted from 100 μl of the sample by addition of 200 μl of methanol (Biosolve Chimie, France), followed by incubation at 72°C for 15 min and addition of 200 μl of cold MilliQ water. Following centrifugation (4,000 *g*, 10 min, 4°C), the supernatants were collected and dried using a GeneVac EZ-2 plus evaporating system (mode: hplc fraction, temperature: 30°C). The dried metabolite extracts were stored at −80°C until metabolomics analyses.

The dried metabolite extracts from the bacteria-conditioned media were derivatized to (MeOx)TMS-derivatives through reaction with 100 μl of 20 mg/ml methoxyamine hydrochloride (Alfa Aesar, A19188) solution in pyridine (Sigma-Aldrich, 437611) for 90 min at 40°C, followed by reaction with 200 μl N-methyl-trimethylsilyl-trifluoroacetamide (MSTFA; Alfa Aesar, A13141) for 12 h at room temperature (Kanani & Klapa, 2007; Kanani *et al*, 2008). GC–MS analysis was performed as previously described (Radic Shechter *et al*, 2021). Briefly, a Shimadzu TQ8040 GC-(triple quadrupole) MS system (Shimadzu Corp.) equipped with a 30 mÅ ~ 0.25 mmÅ ~ 0.25 μm ZB-50 capillary column (7HG-G004-11; Phenomenex) was used. One microlitre of sample was injected in split mode at 250°C using helium as a carrier gas with a flow rate of 1 ml/min. GC oven temperature was held at 100°C for 4 min followed by an increase to 320°C with a rate of 10°C/min, and a final constant temperature period at 320°C for 11 min. The interface and the ion source were held at 280 and 230°C respectively. The detector was operated both in scanning mode, recording in the range of 50–600 m/z, and in MRM mode for specified metabolites. For peak annotation, the GC–MS solution software (Shimadzu Corp.) was utilized. The metabolite identification was based on an in-house database with analytical standards being utilized to define the retention time, the mass spectrum and marker ion fragments for all the quantified metabolites. The metabolite quantification was carried out by integrating the area under the curve of the MRM transition of each metabolite. The data were further normalized to the area under the curve of the MRM transition of ribitol. All peaks were manually checked.

For ^{13}C stable isotope tracing analysis, the dried extracts from the co-cultures were derivatized with 50 μl of 20 mg/ml methoxyamine hydrochloride (Alfa Aesar, A19188) solution in pyridine (Sigma-Aldrich, 437611) for 90 min at 40°C, followed by reaction with 100 μl N-tert-Butyldimethylsilyl-N-methyltrifluoroacetamide +1% tert-Butyldimethylchlorosilane (Sigma-Aldrich, 00942) for 1 h at 60°C. The samples remained at room temperature for 10 h and then analysed by GC–MS. The GC–MS was operated as described above with the following difference: GC oven temperature was held at 100°C for 3 min followed by an increase to 300°C with a rate of 3.5°C/min and a final constant temperature period at 300°C for 10 min. The detector was operated in single ion monitoring (SIM) mode for the ion fragments corresponding to all possible mass isotopologues of the specified metabolites. Mass isotopologue distributions were determined by integrating the area under the curve of the ion fragments. The data were corrected for natural isotope abundance using the Isotope Correction Toolbox (ICT; Jungreuthmayer *et al*, 2016).

Protein extraction

Cell pellets were washed first with water and then with ammonium bicarbonate 50 mM. Pellets were resuspended in RapiGest SF (Waters, 186001860) lysis buffer composed of 1 mg RapiGest ST 0.1%, 4 M urea, 100 mM NH_4HCO_3, 5 mM DTT and protease inhibitor cocktail. Cells were lysed by 3 rounds of bead beating (45 s beating, 1 min rest at 4.5 m/s and 4°C) with zirconia/silica beads. Cell lysates were centrifuged for 1 min at 800 rpm and 4°C, supernatants were recovered and sonicated for 90 s at 4°C (30 s, 3 times at 80% amplitude in 0.5 s on–off cycles with 1 min cooling intervals on ice). Lysate protein concentration was measured by Bradford assay. Protein samples were processed in-solution and all reagents were prepared in 50 mM HEPES (pH 8.5). Cysteines were reduced using dithiothreitol (56°C, 30 min, 10 mM). The sample was then cooled to 24°C and alkylated with iodoacetamide (room temperature, in the dark, 30 min, 10 mM). A novel protocol using paramagnetic beads, termed Single-Pot Solid-Phase-enhanced Sample Preparation (SP3; Hughes *et al*, 2014) was used to prepare the samples for LC–MS/MS. The proteins were digested at 37°C overnight using trypsin (Promega) with an enzyme to protein ratio of 1:50. Peptides were cleaned up using OASIS® HLB μElution Plate (Waters).

Proteomics measurements by LC–MS

Peptides were separated using an ultra-performance liquid chromatography (UPLC) system (nanoAcquity, Waters) fitted with a trapping column (nanoAcquity Symmetry C18, 5 μm, 180 μm × 20 mm) and an analytical column (nanoAcquity BEH C18, 1.7 μm, 75 μm × 200 mm). The outlet of the analytical column was coupled directly to a linear trap quadrupole (LTQ) Orbitrap Velos Pro (Thermo Fisher Scientific) using a Proxeon nanospray source. Solvent A was water with 0.1% formic acid and solvent B was acetonitrile with 0.1% formic acid. The samples were loaded with a constant flow of solvent A at 5 μl/min onto the trapping column. Trapping time was 6 min. Peptides were eluted via the analytical column with a constant flow of 0.3 μl/min. During the elution step, the percentage of solvent B increased in a linear fashion from 3 to 7% in 10 min, then increased to 25% in 100 min and finally to 40% in a further 10 min. The peptides were introduced into the mass spectrometer (Orbitrap Velos, Thermo) via a Pico-Tip Emitter 360 μm OD × 20 μm ID; 10 μm tip (New Objective) and a spray voltage of 2.2 kV was applied. The capillary temperature was set at 300°C. Full scan

MS spectra with mass range 300–1,700 m/z were acquired in profile mode in the FT (Fourier transform) with resolution of 30,000. The filling time was set at a maximum of 500 ms with a limitation of 1.0×106 ions. The most intense ions (up to 15) from the full scan MS were selected for sequencing in the LTQ. A normalized collision energy of 40% was used and the fragmentation was performed after accumulation of 3.0×10^4 ions or after a filling time of 100 ms for each precursor ion (whichever occurred first). MS/MS data were acquired in centroid mode. Only multiply charged (2+, 3+, 4+) precursor ions were selected for MS/MS. The dynamic exclusion list was restricted to 500 entries with a maximum retention period of 30 s and a relative mass window of 10 ppm. Lock mass correction using the ion 445.12003 m/z was applied.

Proteomic data analysis

Rstudio v1.2.5033 and R v3.5.1 were used for data analysis. For protein-SIP analyses, the automated and freely available bioinformatics solution for protein-SIP-based metaproteomic experiments, MetaProSIP (Sachsenberg *et al*, 2015) was used, as well as the workflow engine Konstanz Information Miner (KNIME, v3.6.2) for the analysis of large data sets and production of high-quality visualizations (Berthold *et al*, 2009) and the OpenMS framework (Rost *et al*, 2016). A total of 9 LC–MS/MS raw files were analysed, corresponding to three different labelling conditions: lactose non-labelled, lactose-[^{13}C-galactose] and lactose–[^{13}C-glucose], in triplicates.

For peptide and protein identification with the MetaProSip, raw files were converted to mzML using MSConvertGUI (ProteoWizard Tools, v3.0.18199-78f1f2280; Kessner *et al*, 2008). Subsequently, mzML files were analysed through a 2-step relative KNIME pipeline, where first unlabelled peptides were identified, followed by identification of their labelled isoforms. Data were searched against the yeast (Uniprot proteome UP000002311) and bacteria proteome (Blasche *et al*, 2021). The decoy database contained peptides from human, pig, *Podospermum laciniatum*, *Escherichia coli* and bovine. The unlabelled peptides were identified by the search engine OpenMS MSGFPlusAdapter. The following options were included into the search parameters: a mass error tolerance of 10 ppm for full scan (MS1); trypsin as protease with a maximum of two missed cleavages; carbamidomethylation of cysteines as a fixed modification, oxidation of methionines as a variable modification; a minimal peptide length of 7. After that, through the PeptideIndexer the protein references for all the peptide hits from the idXML files were refreshed and target/decoy information was added to them. The FDR was set to 0.01 on the peptide (PSM) level based on q-value and the maximal number of ambiguous amino acids (AAAs) for matching to the protein database was 3. Furthermore, we used the feature finder tool to detect quantitative features in MS1 data based on information from peptide identifications (derived from MS2 spectra).

Data were then transferred as featureXML-files to the second part of the workflow where the labelled peptides were detected, the related peaks were extracted and eventually the Relative Isotope Abundance (RIA) was calculated through MetaProSip. Proteins were inferred from the peptide identification results by requiring a minimum of two unique peptides per protein. The stability and the data variability were measured by the coefficient of variation (CV) for peptides identified in at least two replicates. Peptides that were identified in only two out of the three replicates and which also had a high CV (> 40%) were filtered out. The median RIA value across the three replicates was computed for each peptide. MLR analyses were performed to elucidate the impact of the AA carbons on each peptide. The standardized values of the amino acid A in peptide P, $stvalues_A^P$, is defined as:

$$stvalues_A^P = \frac{o_A^P * c_A}{len_p}$$

where o_A^P is the number of occurrences of amino acid A in peptide P, c_A is the number of carbons in amino acid A and len_p is the length of peptide P. We then implemented a linear regression model of the normalized values against the RIA to estimate the relative coefficients.

Construction and models simulations

To develop a mechanistic understanding of the interactions between yeast and bacteria we performed *in silico* Flux Balance Analysis (FBA). We initially constructed single-species models from genome assemblies and the two species were then combined to model the syntrophic growth and metabolite accumulation of the co-culture in a given media. The single-species and community models were built with CarveMe (Machado *et al*, 2018) and the Reframed Python package. For the bacterial model, *Lactococcus lactis subsp. Cremoris* MG1363 from the Bigg database (King *et al*, 2016) was used as a reference model. Minor modifications were made to the CDM35-lactose and CDM42-lactose media composition used in simulations to include vitamins and minerals that were essential *in silico*. We first explored the relevance of the presence of lactose and bacterial beta-galactosidase activity by comparing the different FBA growth rate predictions. The same procedure was followed for the co-culture models. In this case, the pre-built CarveMe models were used along with the modified CDM35-lactose medium. All the applied modifications regarding the exchange reactions, metabolites and CDM35-lactose composition were implemented with Python version 3.6. The Smetana tool (Zelezniak *et al*, 2015) was used to map all possible inter-species metabolic exchanges, while the simulation results for each community were summarized as SMETANA score, which estimates the strength of metabolic coupling. For the Smetana community simulation, the same medium composition was used as with Reframed, while water was excluded from calculations.

Data availability

The data sets and code produced in this study are available in the following databases:

- Mass spectrometry proteomics data: ProteomeXchange Consortium via PRIDE (Perez-Riverol *et al*, 2019) PXD019952 (https://www.ebi.ac.uk/pride/archive/projects/PXD019952/)
- Mass spectrometry metabolomics data: EMBL-EBI MetaboLights (Haug *et al*, 2019) MTBLS2063 (https://www.ebi.ac.uk/metabolights/MTBLS2063)

© 2023 The Authors

- Code for metabolic models, simulation results and scripts: GitHub (https://github.com/ChMaga/13C-proteomics).

Expanded View for this article is available online.

Acknowledgements
We thank S. Blasche for kefir *L. lactis* isolates, proteomic core facility (EMBL, Heidelberg) for the protein mass spectrometry, Timo Sachsenberg (Quantitative Biology Center, University of Tübingen, Germany) for support with MetaProSIP, and K. Sadiq for proofreading the manuscript. This project has received funding from the European Union's Horizon 2020 research and innovation programme (Grant agreement no. 866028 (European Research Council (ERC)), and 686070), and UK Medical Research Council (project number MC_UU_00025/11). N.G. was supported by the EMBL Interdisciplinary Postdoc (EIPOD) program under Marie Skłodowska-Curie actions COFUND (grant number 664726).

Author contributions
Natalia Gabrielli: Conceptualization; formal analysis; supervision; investigation; visualization; writing—original draft; writing—review and editing. **Christoniki Maga-Nteve:** Formal analysis; investigation; visualization; methodology; writing—original draft. **Eleni Kafkia:** Formal analysis; investigation; visualization. **Mandy Rettel:** Formal analysis. **Jakob Loeffler:** Investigation. **Stephan Kamrad:** Formal analysis; writing—review and editing. **Athanasios Typas:** Conceptualization; supervision; funding acquisition. **Kiran Raosaheb Patil:** Conceptualization; resources; formal analysis; supervision; funding acquisition; writing—original draft; project administration; writing—review and editing.

In addition to the CRediT author contributions listed above, the contributions in detail are:

NG designed and performed experiments, analysed data and drafted the manuscript. CMN analysed the proteomics data, performed the statistical analysis, constructed the metabolic model and drafted the manuscript. EK oversaw metabolomics experiments and analysed GC–MS data. MR performed the proteomics mass spectrometry analysis. JL created the *gal1Δgal3Δ* yeast mutant strain. SK contributed to manuscript writing and data interpretation. NT contributed to the design and supervision of the project. KRP conceived and oversaw the project, interpreted the data and drafted the manuscript. All authors read, commented on and approved the manuscript.

Disclosure and competing interests statement
The authors declare that they have no conflict of interest.

References

Bastida F, Nicolas C, Moreno JL, Hernandez T, Garcia C (2010) Tracing changes in the microbial community of a hydrocarbon-polluted soil by culture-dependent proteomics. *Pedosphere* 20: 479–485

Benthin S, Nielsen J, Villadsen J (1994) Galactose expulsion during lactose metabolism in Lactococcus lactis subsp. cremoris FD1 due to dephosphorylation of intracellular galactose 6-phosphate. *Appl Environ Microbiol* 60: 1254–1259

von Bergen M, Jehmlich N, Taubert M, Vogt C, Bastida F, Herbst F-A, Schmidt F, Richnow H-H, Seifert
J (2013) Insights from quantitative metaproteomics and protein-stable isotope probing into microbial ecology. *ISME J* 7: 1877–1885

Bernard L, Mougel C, Maron PA, Nowak V, Leveque J, Henault C, Haichar FEZ, Berge O, Marol C, Balesdent J *et al* (2007) Dynamics and identification of soil microbial populations actively assimilating carbon from C-13-labelled wheat residue as estimated by DNA- and RNA-SIP techniques. *Environ Microbiol* 9: 752–764

Berthold MR, Cebron N, Dill F, Gabriel TR, Kötter T, Meinl T, Ohl P, Thiel K, Wiswedel B (2009) KNIME - the Konstanz information miner: version 2.0 and beyond. *ACM SIGKDD Explor Newsl* 11: 26–31

Bianchi F, van't Klooster JS, Ruiz SJ, Poolman B (2019) Regulation of amino acid transport in Saccharomyces cerevisiae. *Microbiol Mol Biol Rev* https://doi.org/10.1128/MMBR.00024-19

Blasche S, Kim Y, Mars RAT, Machado D, Maansson M, Kafkia E, Milanese A, Zeller G, Teusink B, Nielsen J *et al* (2021) Metabolic cooperation and spatiotemporal niche partitioning in a kefir microbial community. *Nat Microbiol* 6: 196–208

Campbell K, Vowinckel J, Mülleder M, Malmsheimer S, Lawrence N, Calvani E, Miller-Fleming L, Alam MT, Christen S, Keller MA *et al* (2015) Self-establishing communities enable cooperative metabolite exchange in a eukaryote. *eLife* 4: e09943

Campbell K, Herrera-Dominguez L, Correia-Melo C, Zelezniak A, Ralser M (2018) Biochemical principles enabling metabolic cooperativity and phenotypic heterogeneity at the single cell level. *Curr Opin Syst Biol* 8: 97–108

Chang CY, Vila JCC, Bender M, Li R, Mankowski MC, Bassette M, Borden J, Golfier S, Sanchez PGL, Waymack R *et al* (2021) Engineering complex communities by directed evolution. *Nat Ecol Evol* 5: 1011–1023

Estrela S, Vila JCC, Lu N, Bajic D, Rebolleda-Gomez M, Chang CY, Goldford JE, Sanchez-Gorostiaga A, Sanchez A (2022) Functional attractors in microbial community assembly. *Cell Syst* 13: 29–42.e27

Ferreira AM, Mendes-Faia A (2020) The role of yeasts and lactic acid bacteria on the metabolism of organic acids during winemaking. *Foods* 9: 1231

Gebreselassie NA, Antoniewicz MR (2015) (13)C-metabolic flux analysis of co-cultures: a novel approach. *Metab Eng* 31: 132–139

Ghosh A, Nilmeier J, Weaver D, Adams PD, Keasling JD, Mukhopadhyay A, Petzold CJ, Martín HG (2014) A peptide-based method for 13C metabolic flux analysis in microbial communities. *PLoS Comput Biol* 10: e1003827

Gietz RD, Schiestl RH (2007) Large-scale high-efficiency yeast transformation using the LiAc/SS carrier DNA/PEG method. *Nat Protoc* 2: 38–41

Giri S, Ona L, Waschina S, Shitut S, Yousif G, Kaleta C, Kost C (2021) Metabolic dissimilarity determines the establishment of cross-feeding interactions in bacteria. *Curr Biol* 31: 5547–5557

Griss J, Jones AR, Sachsenberg T, Walzer M, Gatto L, Hartler J, Thallinger GG, Salek RM, Steinbeck C, Neuhauser N *et al* (2014) The mzTab data exchange format: communicating mass-spectrometry-based proteomics and metabolomics experimental results to a wider audience. *Mol Cell Proteomics* 13: 2765–2775

Guillen MN, Rosener B, Sayin S, Mitchell A (2021) Assembling stable syntrophic Escherichia coli communities by comprehensively identifying beneficiaries of secreted goods. *Cell Syst* 12: 1064–1078

Hackett SR, Zanotelli VR, Xu W, Goya J, Park JO, Perlman DH, Gibney PA, Botstein D, Storey JD, Rabinowitz JD (2016) Systems-level analysis of mechanisms regulating yeast metabolic flux. *Science* 354: aaf2786

Haug K, Cochrane K, Nainala VC, Williams M, Chang J, Jayaseelan KV, O'Donovan C (2019) MetaboLights: a resource evolving in response to the needs of its scientific community. *Nucleic Acids Res* 48: D440–D444

© 2023 The Authors

Henriques SF, Dhakan DB, Serra L, Francisco AP, Carvalho-Santos Z, Baltazar C, Elias AP, Anjos M, Zhang T, Maddocks ODK *et al* (2020) Metabolic cross-feeding in imbalanced diets allows gut microbes to improve reproduction and alter host behaviour. *Nat Commun* 11: 4236

Herbst FA, Bahr A, Duarte M, Pieper DH, Richnow HH, von Bergen M, Seifert J, Bombach P (2013) Elucidation of in situ polycyclic aromatic hydrocarbon degradation by functional metaproteomics (protein-SIP). *Proteomics* 13: 2910–2920

Hughes CS, Foehr S, Garfield DA, Furlong EE, Steinmetz LM, Krijgsveld J (2014) Ultrasensitive proteome analysis using paramagnetic bead technology. *Mol Syst Biol* 10: 757

Hutkins RW, Ponne C (1991) Lactose uptake driven by galactose efflux in Streptococcus thermophilus: evidence for a galactose-lactose antiporter. *Appl Environ Microbiol* 57: 941–944

Jehmlich N, Schmidt F, Hartwich M, von Bergen M, Richnow H-H, Vogt C (2008) Incorporation of carbon and nitrogen atoms into proteins measured by protein-based stable isotope probing (protein-SIP). *Rapid Commun Mass Spectrom* 22: 2889–2897

Jehmlich N, Schmidt F, Taubert M, Seifert J, Bastida F, von Bergen M, Richnow HH, Vogt C (2010) Protein-based stable isotope probing. *Nat Protoc* 5: 1957–1966

Jehmlich N, Vogt C, Lünsmann V, Richnow HH, von Bergen M (2016) Protein-SIP in environmental studies. *Curr Opin Biotechnol* 41: 26–33

Jungreuthmayer C, Neubauer S, Mairinger T, Zanghellini J, Hann S (2016) ICT: isotope correction toolbox. *Bioinformatics* 32: 154–156

Kanani HH, Klapa MI (2007) Data correction strategy for metabolomics analysis using gas chromatography-mass spectrometry. *Metab Eng* 9: 39–51

Kanani H, Chrysanthopoulos PK, Klapa MI (2008) Standardizing GC-MS metabolomics. *J Chromatogr B Analyt Technol Biomed Life Sci* 871: 191–201

Kessner D, Chambers M, Burke R, Agusand D, Mallick P (2008) ProteoWizard: open source software for rapid proteomics tools development. *Bioinformatics* 24: 2534–2536

King ZA, Lu J, Drager A, Miller P, Federowicz S, Lerman JA, Ebrahim A, Palsson BO, Lewis NE (2016) BiGG models: a platform for integrating, standardizing and sharing genome-scale models. *Nucleic Acids Res* 44: D515–D522

Konstantinidis D, Pereira F, Geissen EM, Grkovska K, Kafkia E, Jouhten P, Kim Y, Devendran S, Zimmermann M, Patil KR (2021) Adaptive laboratory evolution of microbial co-cultures for improved metabolite secretion. *Mol Syst Biol* 17: e10189

Leenhouts KJ, Bolhuis A, Ledeboer A, Venema G, Kok J (1995) Production of secreted guar α-galactosidase by *Lactococcus lactis*. *Appl Microbiol Biotechnol* 44: 75–80

Ljungdahl PO (2009) Amino-acid-induced signalling via the SPS-sensing pathway in yeast. *Biochem Soc Trans* 37: 242–247

Machado D, Andrejev S, Tramontano M, Patil KR (2018) Fast automated reconstruction of genome-scale metabolic models for microbial species and communities. *Nucleic Acids Res* 46: 7542–7553

Machado D, Maistrenko OM, Andrejev S, Kim Y, Bork P, Patil KR, Patil KR (2021) Polarization of microbial communities between competitive and cooperative metabolism. *Nat Ecol Evol* 5: 195–203

Morishita T, Yajima M (1995) Incomplete operation of biosynthetic and bioenergetic functions of the citric acid cycle in multiple auxotrophic lactobacilli. *Biosci Biotechnol Biochem* 59: 251–255

Mulleder M, Capuano F, Pir P, Christen S, Sauer U, Oliver SG, Ralser M (2012) A prototrophic deletion mutant collection for yeast metabolomics and systems biology. *Nat Biotechnol* 30: 1176–1178

Paczia N, Nilgen A, Lehmann T, Gatgens J, Wiechert W, Noack S (2012) Extensive exometabolome analysis reveals extended overflow metabolism in various microorganisms. *Microb Cell Fact* 11: 122

Perez-Riverol Y, Csordas A, Bai JW, Bernal-Llinares M, Hewapathirana S, Kundu DJ, Inuganti A, Griss J, Mayer G, Eisenacher M *et al* (2019) The PRIDE database and related tools and resources in 2019: improving support for quantification data. *Nucleic Acids Res* 47: D442–D450

Pino A, Russo N, Solieri L, Sola L, Caggia C, Randazzo CL (2022) Microbial consortia involved in traditional Sicilian sourdough: characterization of lactic acid bacteria and yeast populations. *Microorganisms* 10: 283

Ponomarova O, Gabrielli N, Sevin DC, Mulleder M, Zimgibl K, Bulyha K, Andrejev S, Kafkia E, Typas A, Sauer U *et al* (2017) Yeast creates a niche for symbiotic lactic acid bacteria through nitrogen overflow. *Cell Syst* 5: 345–357

Pontrelli S, Szabo R, Pollak S, Schwartzman J, Ledezma-Tejeida D, Cordero OX, Sauer U (2022) Metabolic cross-feeding structures the assembly of polysaccharide degrading communities. *Sci Adv* 8: eabk3076

Radajewski S, Ineson P, Parekh NR, Murrell JC (2000) Stable-isotope probing as a tool in microbial ecology. *Nature* 403: 646–649

Radic Shechter K, Kafkia E, Zirngibl K, Gawrzak S, Alladin A, Machado D, Luchtenborg C, Sevin DC, Brugger B, Patil KR *et al* (2021) Metabolic memory underlying minimal residual disease in breast cancer. *Mol Syst Biol* 17: e10141

Rost HL, Sachsenberg T, Aiche S, Bielow C, Weisser H, Aicheler F, Andreotti S, Ehrlich HC, Gutenbrunner P, Kenar E *et al* (2016) OpenMS: a flexible open-source software platform for mass spectrometry data analysis. *Nat Methods* 13: 741–748

Sachsenberg T, Herbst FA, Taubert M, Kermer R, Jehmlich N, von Bergen M, Seifert J, Kohlbacher O (2015) MetaProSIP: automated inference of stable isotope incorporation rates in proteins for functional Metaproteomics. *J Proteome Res* 14: 619–627

Sauro HM (2017) Control and regulation of pathways via negative feedback. *J R Soc Interface* 14: 20160848

Schwechheimer SK, Becker J, Wittmann C (2018) Towards better understanding of industrial cell factories: novel approaches for C-13 metabolic flux analysis in complex nutrient environments. *Curr Opin Biotechnol* 54: 128–137

Stovicek V, Borja GM, Forster J, Borodina I (2015) EasyClone 2.0: expanded toolkit of integrative vectors for stable gene expression in industrial Saccharomyces cerevisiae strains. *J Ind Microbiol Biotechnol* 42: 1519–1531

Vogt C, Lueders T, Richnow HH, Kruger M, von Bergen M, Seifert J (2016) Stable isotope probing approaches to study anaerobic hydrocarbon degradation and degraders. *J Mol Microbiol Biotechnol* 26: 195–210

Wiechert W (2001) C-13 metabolic flux analysis. *Metab Eng* 3: 195–206

Winzeler EA, Shoemaker DD, Astromoff A, Liang H, Anderson K, Andre B, Bangham R, Benito R, Boeke JD, Bussey H *et al* (1999) Functional characterization of the S-cerevisiae genome by gene deletion and parallel analysis. *Science* 285: 901–906

Xiao T, Khan A, Shen Y, Chen L, Rabinowitz JD (2022) Glucose feeds the tricarboxylic acid cycle via excreted ethanol in fermenting yeast. *Nat Chem Biol* 18: 1380–1387

Yu JSL, Correia-Melo C, Zorrilla F, Herrera-Dominguez L, Wu MY, Hartl J, Campbell K, Blasche S, Kreidl M, Egger AS *et al* (2022) Microbial

 © 2023 The Authors

communities form rich extracellular metabolomes that foster metabolic interactions and promote drug tolerance. *Nat Microbiol* 7: 542–555

Zelezniak A, Andrejev S, Ponomarova O, Mende DR, Bork P, Patil KR (2015) Metabolic dependencies drive species co-occurrence in diverse microbial communities. *Proc Natl Acad Sci USA* 112: 6449–6454

Zelezniak A, Vowinckel J, Capuano F, Messner CB, Demichev V, Polowsky N, Mülleder M, Kamrad S, Klaus B, Keller MA *et al* (2018) Machine learning predicts the yeast metabolome from the quantitative proteome of kinase knockouts. *Cell Syst* 7: 269–283.e66

License: This is an open access article under the terms of the Creative Commons Attribution License, which permits use, distribution and reproduction in any medium, provided the original work is properly cited.

Expanded View Figures

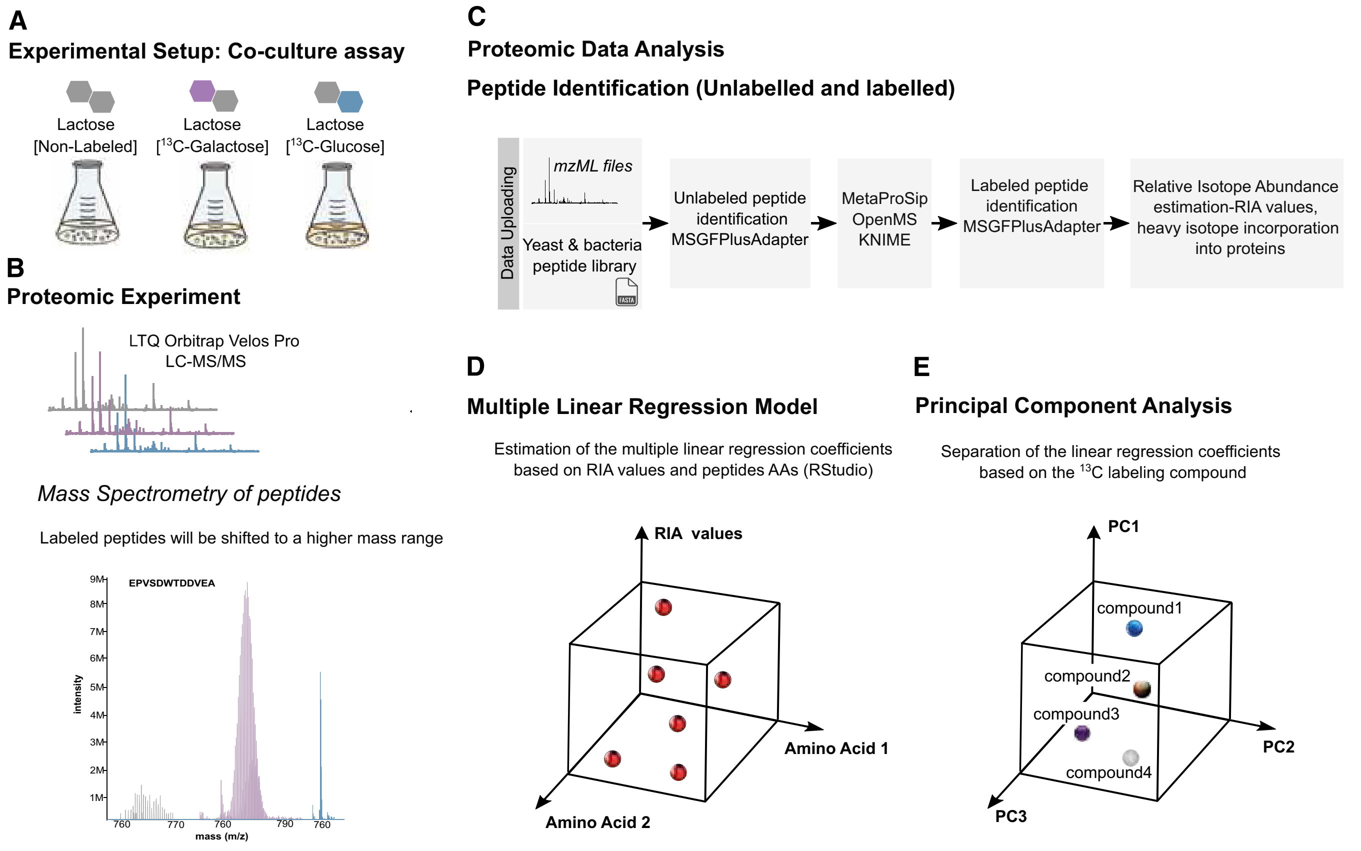

Figure EV1. Detailed workflow diagrams.

A Proteomics samples were collected from co-cultures grown in CDM35-lactose media with three different labelling regimes: non-labelled lactose, lactose with the galactose moiety labelled and lactose with the glucose moiety labelled.

B Tryptic peptides from all three labelling regimes were analysed on an LTQ Orbitrap Velos Pro instrument and the mass shift and relative isotope abundance values were determined from MS1 spectra.

C Overview of data analysis pipeline.

D Multiple linear regression (MLR) was used to determine the contribution of individual amino acids to peptide labelling in the different conditions.

E PCA was used for a global comparison of MLR coefficient profiles across different labelling regimes.

 © 2023 The Authors

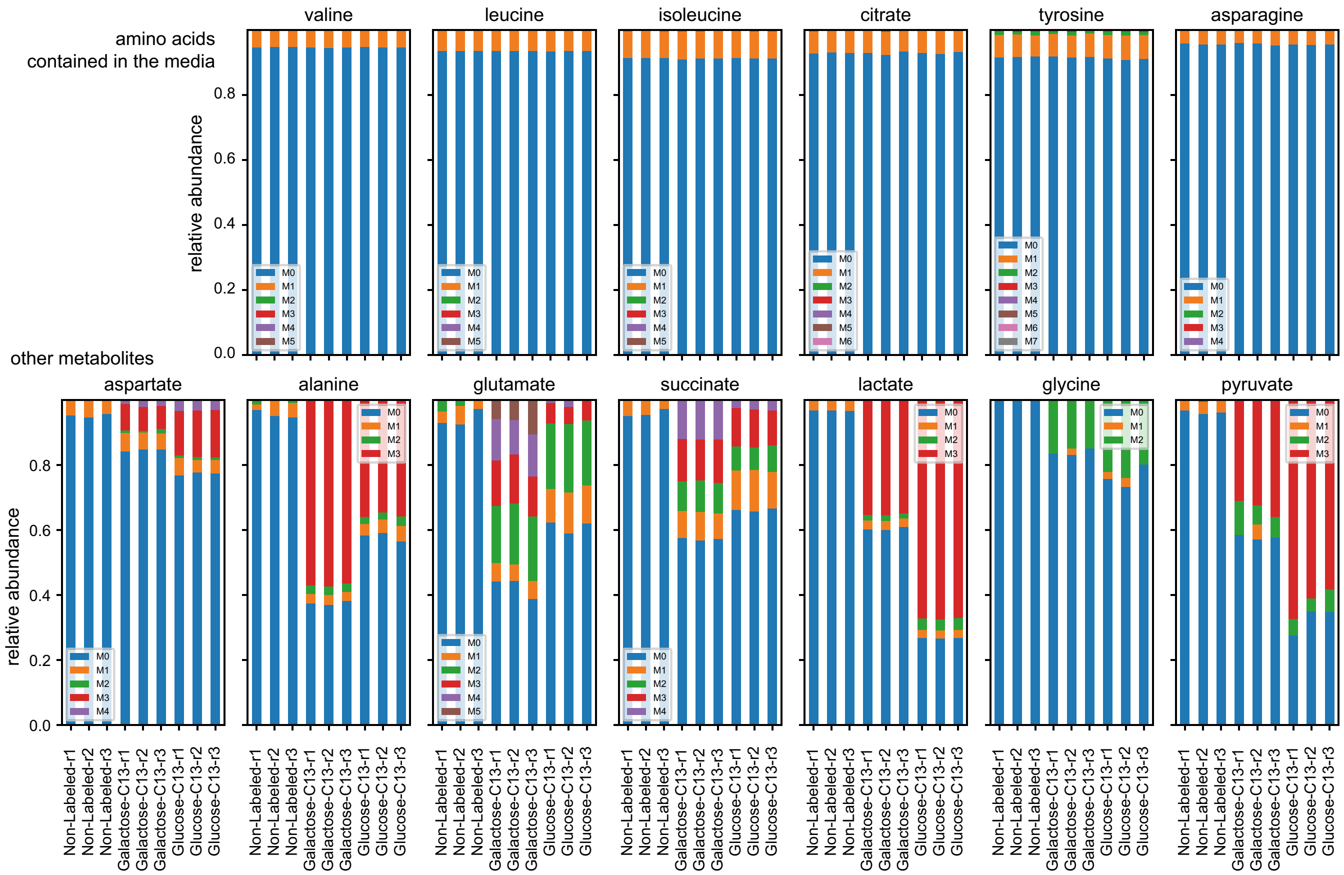

Figure EV2. Illustration of labelling states of extracellular metabolites in culture supernatant under three different labelling regimes.

Three biological replicates are shown individually.

© 2023 The Authors

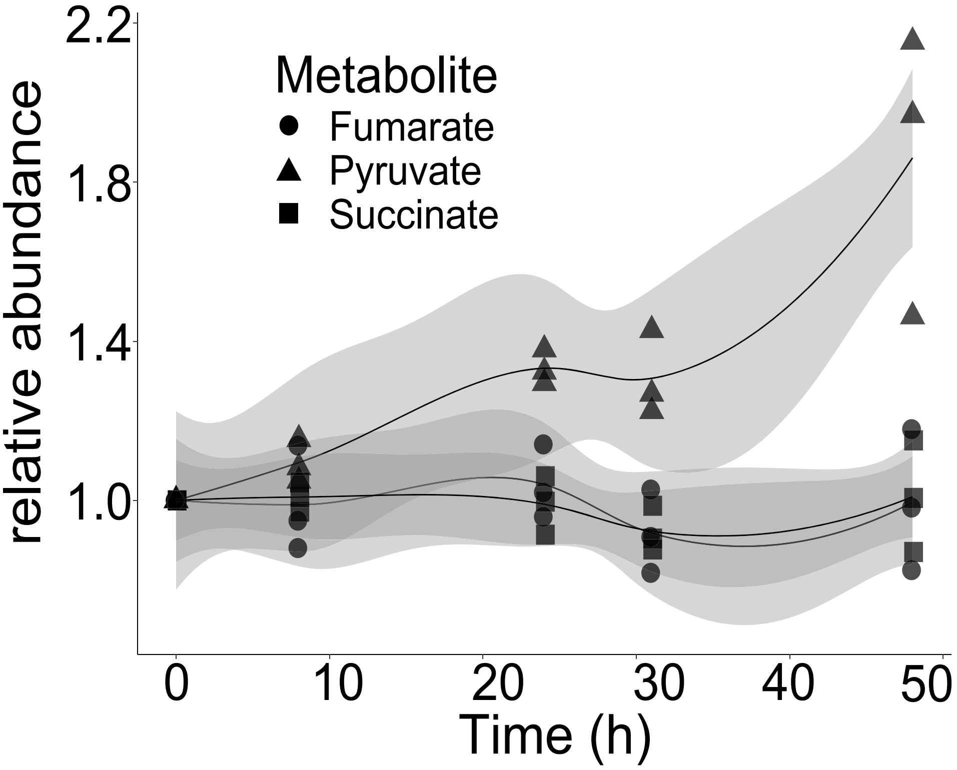

Figure EV3. Concentration dynamics of three metabolites during growth of *S. cerevisiae* on bacteria-conditioned media.

(n = 3 biological replicates)

Figure EV4. Composition of CDM42- and CDM35-lactose media.

CDM42-lactose contains many amino acids, supports growth of *L. lactis* monocultures and was used to generate bacteria-conditioned medium. CMD35-lactose contains fewer amino acids, does not support *L. lactis* growth and was used for co-culture experiments as it results in a mutualistic co-culture where neither species can grow alone.

 © 2023 The Authors

	Component	Concentration, g/L CDM42-lactose	CDM35-lactose
**C-source**	Lactose	40	40
**Amino Acids**	L-Glutamic acid	0.6	**0**
	L-Aspartic acid	0.05	**0**
	L-Glutamine	0.6	**0**
	L-Serine	0.5	**0**
	L-Threonine	0.5	**0**
	L-Alanine	0.4	**0**
	L-Glycine	0.3	**0**
	L-Arginine	0.72	0.72
	L-Asparagine	0.5	0.5
	L-Histidine	0.17	0.17
	L-Isoleucine	0.24	0.24
	L-Leucine	1	1
	L-Methionine	0.125	0.125
	L-Tyrosine	0.3	0.3
	L-Valine	0.7	0.7
**Vitamins**	Biotin	0.006	0.006
	Ca pantothenate	0.0012	0.0012
	Folic acid	0.00056	0.00056
	myo-Inositol	0.002	0.002
	Nicotinic acid	0.0009	0.0009
	p-Aminobenzoic acid	0.000056	0.000056
	Pyridoxine HCl	0.0048	0.0048
	Riboflavin	0.0009	0.0009
	Thiamine HCl	0.00056	0.00056
**Inorganic Salts**	$FeSO_4 \cdot 7H_2O$	0.005	0.005
	K_2HPO_4	6.48	6.48
	KH_2PO_4	3.12	3.12
	$MgCl_2$	0.3864	0.3864
	NaCl	3	3
	$ZnSO_4$	0.005	0.005
	K_2SO_4	0.023	0.023
	Boric acid	0.00075	0.00075
	$CaCl_2$	0.03	0.03
	$CoCl_2 \cdot 6H_2O$	0.00019	0.00019
	$CuSO_4$	0.00012	0.00012
	KI	0.00011	0.00011
	$MnSO_4 \cdot H_2O$	0.00034	0.00034
	$(NH_4)6Mo_7O_{24} \cdot 4H_2O$	0.00019	0.00019
**Other**	Ammonium citrate dibasic	1.69	1.69
	Citric acid$\cdot H_2O$	0.003	0.003
	L-Glutathione reduced	0.015	0.015
	pH	7	7

Figure EV4.
